# Indian Journal of Plastic Surgery in 2025

**DOI:** 10.1055/s-0046-1818624

**Published:** 2026-03-25

**Authors:** Maneesh Singhal

**Affiliations:** 1 Department of Plastic Surgery, All India Institute of Medical Sciences, New Delhi, India

Plastic surgery in India has seen remarkable growth over the past decade, evolving into a dynamic and innovation-driven clinical specialty. Advances in reconstructive and aesthetic techniques, improvements in training pathways, and increasing engagement in research have collectively expanded both the scope and impact of plastic surgery practice across the country. From complex microsurgical reconstructions to advanced aesthetic and gender-affirming procedures, we continue to contribute meaningful innovations that translate into better patient care and improved quality of life.

Within this rapidly progressing landscape, I see *Indian Journal of Plastic Surgery (IJPS)* as a central academic platform for the specialty. The journal has consistently provided an important forum for students, trainees, practicing surgeons, and researchers to share original research, technical refinements, and clinically relevant experiences. Beyond documenting innovation, I believe IJPS plays a key role in bridging research and day-to-day clinical practice. By prioritizing studies rooted in real-world patient care, the journal supports evidence-based decision-making and helps surgeons achieve reliable functional and aesthetic outcomes.

I see 2025 as an important milestone in the journal's journey. A clear rise in its impact factor reflects not only growing readership but also increased citation rates and wider recognition within the scientific community. In my assessment, this progress highlights the maturation of IJPS as a credible and influential voice in global plastic surgery literature. This upward trajectory reflects a deliberate focus on clinically relevant research, the expansion of its digital footprint, the adoption of environmentally conscious practices, the streamlining of editorial workflows, and the development of a strong editorial team, all while maintaining high ethical standards. These efforts ensure that IJPS remains a dependable resource for surgeons seeking both practical guidance and scientific insight.

Among the many high-quality contributions published this year, I have chosen to highlight a few articles that stand out for their clinical relevance and scholarly merit. Together, they reflect how contemporary Indian plastic surgery research is closely aligned with real patient needs. Several studies explore innovative reconstructive and aesthetic techniques that broaden the therapeutic options available to surgeons. Work on orbital reconstruction in rhino-orbital-cerebral mucormycosis, the use of CAD-CAM technology for maxillary reconstruction, and a standardized approach to hyaluronic acid fillers in nonsurgical rhinoplasty demonstrate how thoughtful innovation can lead to safer procedures, better tissue outcomes, and improved aesthetics. 1 2 3 These studies illustrate the effective integration of anatomical knowledge, sound surgical judgment, and patient-centered care.

I found two studies featuring breast surgery particularly impactful. One evaluates quality-of-life improvement following lymphovenous anastomosis in patients with breast cancer-related lymphedema, highlighting the importance of functional recovery and long-term survivorship. The companion article introduces a novel technique aimed at preserving upper-pole fullness after breast reduction, addressing a common aesthetic concern and refining existing operative strategies. 4 5 Together, these studies reflect what I see as a growing emphasis on outcome-driven practice, where success is judged not only by technical execution but also by patient-reported benefit. 6 7 8

I felt that the nationwide survey on work-related musculoskeletal disorders among practicing plastic surgeons deserved special attention, as it highlights an important aspect of surgeon well-being. 9 While specific to plastic surgery, its findings have broader implications, underscoring the need for ergonomic awareness, preventive strategies, and sustainable surgical practice. In parallel, the “Eklavya—Do-It-Yourself” microsurgical training model presents an innovative and accessible approach to skill development, reinforcing the value of simulation-based learning in enhancing surgical precision and patient safety. 10

The final highlighted article reflects the long-term educational vision of the Association of Plastic Surgeons of India. By evaluating the APSI Postgraduate Medical Education (APSI-PGME) program, the authors outline a structured initiative aimed at strengthening postgraduate training, standardizing educational quality, and producing clinically competent surgeons for the future. 11 I firmly believe that high-quality education remains inseparable from high-quality patient care, and initiatives such as APSI-PGME represent an essential investment in the specialty's continued growth.

Taken together, these selected articles portray a specialty that is advancing through innovation, education, and clinical accountability. I believe IJPS occupies a pivotal position within this ecosystem by encouraging scholarly exchange and facilitating the adoption of evidence-based practices that directly influence surgical outcomes.

Looking forward, I believe the future of plastic surgery in India will be shaped by closer integration of clinical research, technology-enabled training, and multidisciplinary collaboration. As patient expectations continue to rise and surgical challenges grow more complex, journals such as IJPS will play an increasingly important role in guiding best practices, disseminating validated innovations, and fostering a culture of continuous improvement. By supporting clinically meaningful research and nurturing young investigators, IJPS is well positioned to influence national standards of care while contributing to the global discourse in reconstructive and aesthetic surgery.

I take pride in the fact that India hosts a journal that not only advances super-specialty care within the country but also shares indigenous innovations and best practices with the global community.
